# Next-generation sequencing-based detection in a breast MMPMN patient with EGFR T790M mutation: a rare case report and literature review

**DOI:** 10.3389/fonc.2023.1204041

**Published:** 2023-07-24

**Authors:** Huiyun Lv, Aijuan Tian, Shanshan Zhao, Jinbo Zhao, Chen Song

**Affiliations:** ^1^ Department of Oncology, The Second Hospital of Dalian Medical University, Dalian, China; ^2^ Department of Nuclear Medicine, The Second Hospital of Dalian Medical University, Dalian, China

**Keywords:** EGFR T790M mutation, multiple primary malignant neoplasms, next-generation sequencing, osimertinib, breast cancer

## Abstract

Multiple primary malignant neoplasms (MPMNs) are difficult to identify from the metastasis or recurrence of malignant tumors. Additionally, the genetic mutations in each primary tumor vary from each other; therefore, it is critical to explore potential abnormal genes. Next-generation sequencing (NGS) technology has emerged as a reliable approach for detecting mutated genes in primary tumors and can provide several targeted therapeutic options for patients with MPMNs. Here, we report a case of metachronous multiple primary malignant neoplasm (MMPMN) patient with primary ovarian and breast cancer. Targeted NGS genetic profiling revealed a rare EGFR T790M mutation in this patient’s primary breast tumor tissue, which has only been reported previously in breast cancer (BC). Based on the NGS results, osimertinib was recommended for this patient. Although this patient did not receive osimertinib because of gastrointestinal hemorrhage, this case highlights the significance of NGS technology in the diagnosis and treatment of MPMNs.

## 1 Introduction

Multiple primary malignant neoplasms (MPMNs) present an increasing incidence rate owing to the detection of early stages of cancer and the development of effective therapeutic strategies (1). MPMNs are defined as two or more unrelated primary malignant tumors that originate from different organs and occur simultaneously or one after the other (2). MPMNs are classified into two subtypes: synchronous multiple primary malignant neoplasms (SMPMNs) and metachronous multiple primary malignant neoplasms (MMPMNs). SMPMNs are defined as secondary and primary cancers that occur simultaneously or within 6 months of the first primary cancer. If the interval time is more than 6 months, such tumors are called MMPMNs (3). To date, the prevalence of MPMNs is approximately 0.7%–11% and reports of MPMNs mainly focus on lung cancer and gastrointestinal tumors (4). There are few reports on female patients with multiple primary malignant neoplasms of breast cancer or genital malignancies.

The epidermal growth factor receptor (EGFR) is one of the major oncogenes identified in a variety of human cancers, including breast cancer (5), and is one of the most common driver genes in non-small cell lung cancer (NSCLC) (6). Although tyrosine kinase inhibitor (TKI) targeting EGFR have shown good initial response in NSCLC with mutated EGFR genes, the development of acquired resistance remains inevitable and has emerged as a major limitation of EGFR-targeted therapies with TKIs (7), with disease progression 10–12 months after treatment initiation in most patients (8). In approximately 60% of cases, the most frequent mechanism of acquired resistance is secondary T790M mutation in exon 20, and osimertinib is the standard second-line therapy (9). However, 2% of patients harbor either somatic or germline T790M mutations before any exposure to EGFR-TKIs, resulting in primary resistance (10). In contrast, EGFR mutations have been reported to be rare in human BC, whereas EGFR overexpression and/or amplification have been shown to occur frequently in human breast cancer (5, 11). EGFR T790M mutations are thought to be rare (12). In recent years, next-generation sequencing (NGS) has become available for distinguishing between multiple primary cancers and primary cancer metastasis in MPMNs and has facilitated the identification of targetable gene mutations in different primary tumors in patients. Various clinical studies have shown the promise of site-specific treatment and targeted therapy based on NGS testing results (13). Therefore, NGS containing related genes of great significance for the diagnosis and precise treatment of cancer is urgently needed and warrants further clinical investigation.

Here, we present a rare case of a 59-year-old female patient with MMPMNs harboring a pathogenic EGFR T790M mutation in breast cancer primary sites by NGS genetic testing.

## 2 Case presentation

A 59-year-old female without a genetic family history was diagnosed with high-grade left ovarian serous papillary carcinoma in June 2015. However, her detailed medical history revealed no family history of cancer or exposure to environmental risk factors. Subsequently, the patient underwent radical hysterectomy, bilateral salpingo-oophorectomy, partial rectotomy, and pelvic lymphadenectomy. Postoperative pathological evaluation of the resected tumoral tissues indicated high-grade serous papillary carcinoma of the left ovary, invading the abdomen and pelvic cavity. Metastatic lesions included the muscular wall of the uterine body, left fallopian tube, spleen, omentum greater, part of the diaphragm and mass, appendix, and part of the muscular layer of the rectum with the intestinal wall. Immunohistochemistry (IHC) was positive for estrogen receptor (ER), progesterone receptor (PR), high-grade cervical squamous intraepithelial lesion marker p16, and tumor marker p53, and the pathological stage was IIIC. After surgery, she received systemic chemotherapy with the TC protocol (taxol 300 mg (175 mg/m^2^) in combination with carboplatin 450 mg (dosed by AUC), given every 21 days); however, the number of cycles was unknown. In April 2017, the patient underwent another abdominal tumor resection to relieve the symptoms caused by the tumors compressing the abdominal organs. Histopathology and immunochemistry confirmed metastatic adenocarcinoma of the abdominal cavity, consistent with high-grade serous carcinoma metastasis in the pelvic cavity.

**Figure 1:**
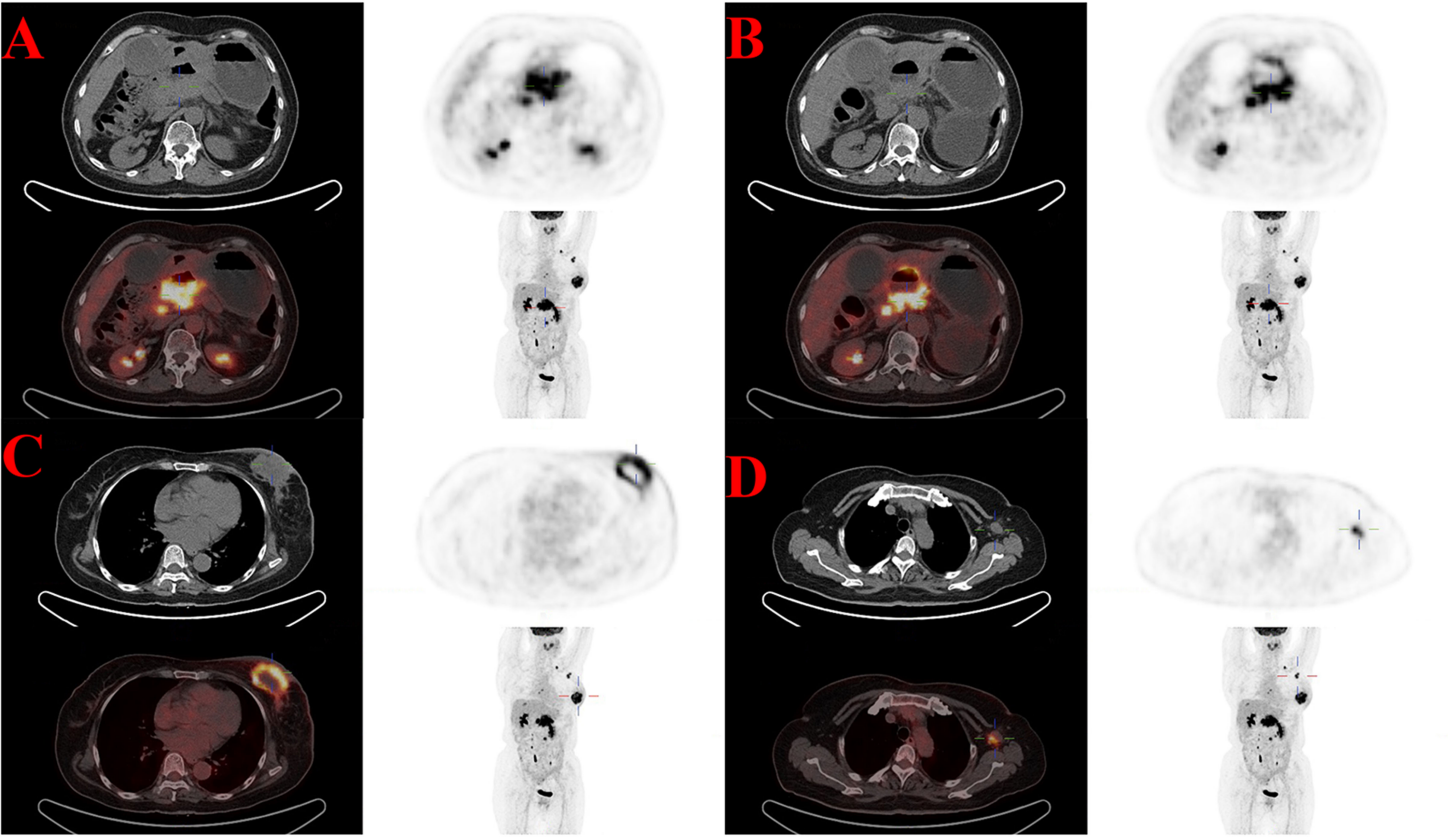
High uptake of 18F-FDG in the known lesions with increasing radioactivity uptake of 18F-FDG PET/CT in September 2022. **(A)** The hepatopancreatic lesions (SUVmax = 19.8). **(B)** The dilatation of the distal main pancreatic duct and the intrahepatic bile duct system. **(C)** The breast lesion (SUVmax = 12.9). **(D)** Multiple axillary lymph nodes lesions (SUVmax = 10.4).

Seven years after the diagnosis of ovarian cancer, the patient began to feel ill, and the main symptoms included abdominal distension and mild jaundice. She underwent whole-body Fluorine-18 fluorodeoxyglucose positron emission tomography/computed tomography (18F-FDG PET/CT) in September 2022. High uptake of 18F-FDG was noted within the known lesions in the hepatopancreatic lesions with increasing radioactivity uptake (**Figure 1A**), which was new and increased in scope from March 2017. All of these were considered malignant, and metastatic tumors led to dilatation of the distal main pancreatic duct and the intrahepatic bile duct system (**Figure 1B**). Additionally, multiple lymph nodes with high FDG uptake were observed throughout the body, indicating metastasis. An avid enhancing soft tissue density lesion with the size of approximately 5.3 ∗ 4.3 cm was noted incidentally in the left breast, showing an uneven increase in radioactive uptake (SUVmax = 12.9) (**Figure 1C**). The adjacent skin was diffusively thickened, showing a slightly increased radioactive uptake (SUVmax = 3.3). Radioactive uptake in the right mammary gland is uniform. Multiple lymph nodes with increased radioactive uptake were observed in the left axillary region (**Figure 1D**). 18F-FDG PET/CT suggested that further pathological examination is warranted for breast lesions with high FDG uptake. The patient received the breast enhanced magnetic resonance imaging (MRI) examination at the same time. The results indicated that the left breast solid mass with skin thickening (BI-RADS grade 5) was 5.3 ∗ 6.0 ∗ 5.3 cm in size, and the left axillary lymph nodes were enlarged with a maximum of 2.1 ∗ 1.1 cm. In addition, there were some enhanced nodules in the right breast (BI-RADS 4A) (**Supplementary Figure S1**).

**Figure 2:**
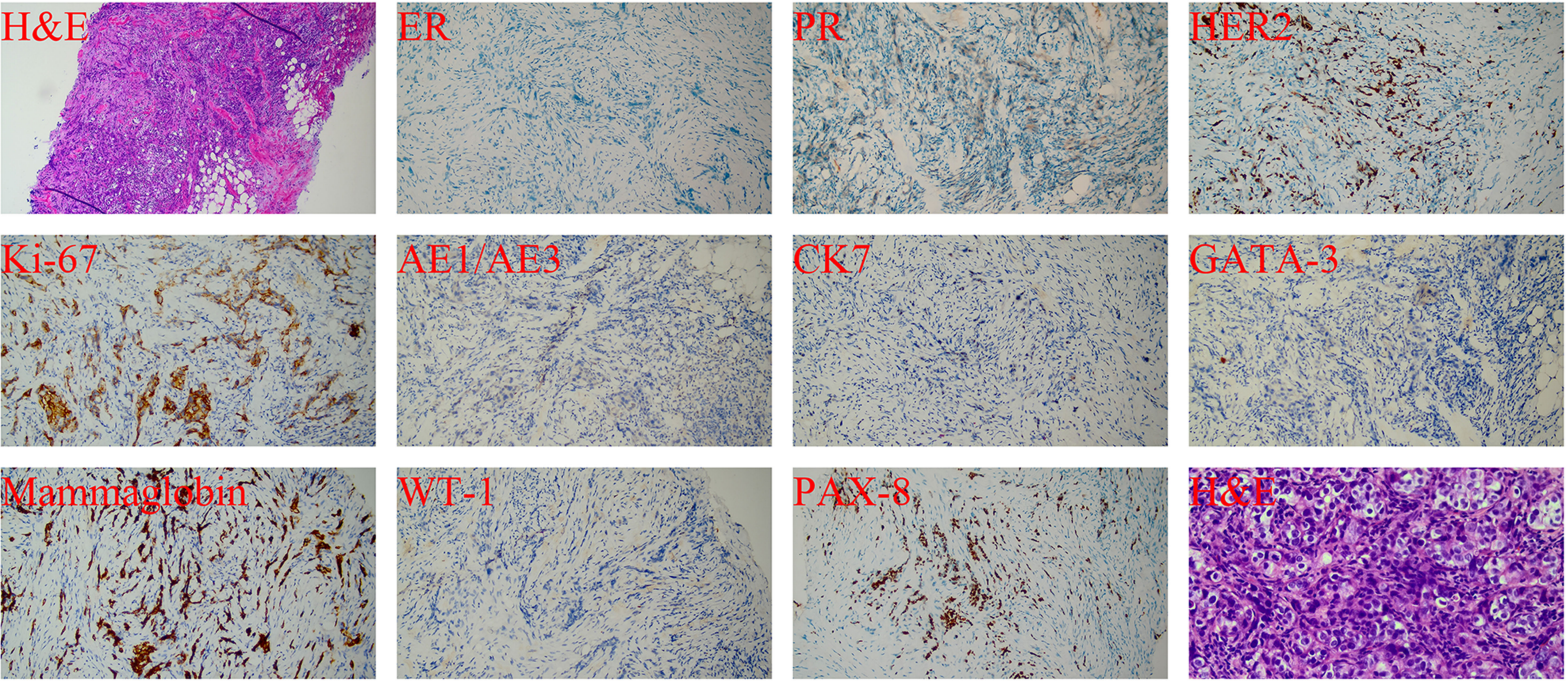
H&E(100× &400×) and IHC(200×) staining of the breast tissue in September 2022.

Considering a primary breast tumor with multiple metastases, ultrasound-guided core needle biopsy of the left breast mass and lymph nodes was performed in September 2022. The diagnosis of invasive ductal carcinoma of the left breast was confirmed by the pathological evaluation of the mass. To exclude breast metastasis, we compared the results with those of previous surgical pathology and confirmed primary breast cancer. Moreover, an invasive ductal carcinoma metastasis was observed in the left axillary lymph nodes. Finally, the pathological staging was determined to be IIB (cT2N1M0). The pathological results (**Figure 2**) were as follows: hematoxylin and eosin (H&E) staining revealed a histological pattern of adenocarcinoma. IHC staining showed that both ER and PR were negative, and human epidermal growth factor receptor 2 (HER2) was moderately positive without amplification as detected by FISH. The tumor proliferation marker antigen Ki-67 was 35% positive, and others were tumor marker p53 (diffuse,+), intestinal adenocarcinoma marker cytokeratin7 (CK7,+), ovarian cancer marker Wilms tumor 1 (WT1, −), ovarian clear cell carcinoma marker, and Paired Box 8 (PAX8,−) marker for renal, Müllerian, and thyroid carcinomas. Computed tomography (CT) of the chest, abdomen, and pelvis was performed to rule out metastatic disease. Fortunately, no breast cancer-related metastases were detected. Finally, the patient was diagnosed with MMPMNs, including left breast invasive ductal carcinoma with left axillary lymph node metastasis and high-grade left ovarian serous papillary carcinoma with extensive abdominal and pelvic metastases. For a more detailed evaluation of the left breast lesion, contrast-enhanced breast MRI was performed, which displayed that left breast solid mass (BI-RADS Grade 5) 5.3 ∗ 6.0 ∗ 5.3 cm in size. In addition, multiple swollen lymph nodes in the left axilla were observed, with a maximum of 2.1 ∗ 1.1 cm indicating metastases.

To further explore a more efficient therapeutic strategy for this MMPMN patient, freshly collected plasma and formalin-fixed, paraffin-embedded (FFPE) primary ovarian and breast tumor tissues were subjected to NGS in October 2022. Targeted NGS of 425 cancer-related genes (**Supplementary Table S1**) was performed at Nanjing Geneseeq Technology Inc., approved by the College of American Pathologists (CAP) and Clinical Laboratory Improvement Amendments (CLIA). An EGFR p.T790M (c.2369C>T) mutation was revealed at a mutant allele frequency (MAF) of 1.59% in primary breast cancer tissue alone, compared to the primary ovary tissue and the plasma sample, which identified the EGFR T790M mutation as a new tumor-initial driver event in BC. Detailed results of the genetic alterations are shown (**Supplementary Table S2**). In particular, this patient did not have BRCA1–2 mutations, which, to some extent, ruled out a link between the patient’s breast cancer and her previous ovarian cancer.

As such, our doctors communicated fully with the patient and her family, who were adequately informed and agreed to the treatment plan. In October 2022, the therapeutic regimen was planned as chemotherapy (docetaxel 120 mg/m^2^ in combination with carboplatin 600 mg/m^2^; administered every 21 days for six cycles), followed by osimertinib-targeted therapy, which is a promising, orally available, third-generation mutation-specific EGFR TKI for the treatment of EGFR T790M resistance mutation-positive NSCLC. However, no treatment options for breast cancer patients with the EFGR T790M mutation have been reported in the literature. Unfortunately, the patient died after the first cycle of chemotherapy (November 2022) due to worsening gastrointestinal hemorrhage and hypovolemic shock with disease progression.

## 3 Discussion

In recent years, identifying genetic mutations in EGFR has resulted in significant changes in the diagnosis and management of cancer (14). Mutations in the EGFR gene regulate cell proliferation and differentiation; hence, it is important to promote the development of cancer (15). EGFR gene is located on chromosome 7p11.2, contains 28 exons, and encodes including a cytoplasmic domain (also called the tyrosine kinase domain), which is responsible for the phosphorylation of its downstream targets and self-regulation (15). EGFR-TKIs are currently the first-line treatment for cancer patients with EGFR-sensitive mutations (16). EGFR mutations are present in solid tumors with a variety of mutation types that may affect the response to EGFR-TKIs. Different mutation types increase the activity of EGFR and activate different downstream signaling pathways (17); this cascade relates RTK activity to increased proliferation, motility, migration, survival, and anti-apoptotic cellular responses and facilitates the genesis and development of cancer (18). The most common alterations are deletions in exon 19 (ex19del, about 44%) and point mutations in exon 21 (L858R, about 41%), known as common mutations or classical mutations, which are considered sensitive to treatment with TKIs (19).

Patients with EGFR-TKIs as first-line treatment have an average progression-free survival (PFS) of 10–12 months (20); however, acquired resistance is inevitable. Secondary resistance mutations in the tyrosine kinase domain develop in up to 60% of patients with NSCLC receiving EGFR-TKIs, most commonly the secondary mutation of resistance p.Thr790Met (T790M), resulting from a gatekeeper mutation in exon20 of EGFR (21). This is referred to as a “gatekeeper” mutation because the 790 residue is in a key location at the entrance to the hydrophobic pocket of the adenosine triphosphate (ATP)-binding cleft (22). The T790M mutation results in a conformational change in the ATP-binding pocket and increases the affinity for ATP in the ATP-binding domain of EGFR. As a result, T790M causes steric hindrance of the binding to their connected ATP binding site on EGFR of an ATP-competitive kinase inhibitor (first- and second-generation TKIs), but irreversible inhibitors (third generation TKIs) overcome this resistance simply through covalent binding (23).

Compared to other selective third-generation mutation-specific EGFR TKIs inhibiting the T790M mutation, osimertinib has shown great superiority and was approved by the US Food and Drug Administration (FDA) as a competitive inhibitor of EGFR T790M-positive NSCLC patients progressing following EGFR-TKI therapy (24). More precisely, osimertinib irreversibly and covalently binds to the cysteine-797 residue in the ATP-binding pocket of EGFR, regardless of the hindering of T790M. Furthermore, because osimertinib creates an irreversible link with the ATP pocket of EGFR, it can overcome the increased affinity of ATP determined by the T790M mutation (23). EGFR T790M mutation in tumors is common and almost entirely presents as a secondary mutation, especially in NSCLC. However, it is extremely rare in breast tumors, and there are limited data supporting the role of EGFR T790M-directed agents in BC. To the best of our knowledge, only three existing BC cases with EGFR T790M positivity have been reported in a 2015 Norwegian clinical study, all of which were primary unilateral breast cancer (UBC) (5).

With the development of precision therapy, the model of targeted therapy guided by comprehensive gene testing is gradually mature, and NGS technology has been widely accepted in the aspects of disease diagnosis, targeted therapy, efficacy evaluation, drug resistance monitoring and other applications (13). Currently, the importance of NGS has been highlighted for discovering rare genetic alterations to guide disease prevention and to improve treatment decision-making and the use of targeted therapy. With the popularity of NGS, if a patient’s economic condition permits, clinicians can recommend that the patient accepts NGS as early as possible, especially lung cancer (13–25). It can determine whether the patient has a target for follow-up immunotargeted therapy or immunochemotherapy combined therapy. In addition, at the critical point of disease recurrence or metastasis, NGS can effectively provide a new direction for therapy, thereby bringing new hope to patients (26). In terms of which type of NGS to perform, clinicians should advise the patient to make a discretionary choice based on each patient’s own condition and financial situation (27). In the present case, a pathogenic EGFR T790M mutation was identified in an MMPMN patient’s primary breast sample alone by NGS, indicating the driving role of this mutation in BC. The benefit of NGS in identifying novel potential molecular targets for subsequent treatment has been confirmed (28). In addition, NGS can not only provide more detailed information for diagnosis and treatment decision making, but also reveal the efficacy and monitor drug resistance during targeted therapy.

The limitations of the single case presentation in this study should be noted. The patient died because of severe gastrointestinal bleeding and hypovolemic shock before receiving osimertinib. Therefore, the antitumor effect of ositinib could not be reflected in the treatment of this patient; however, our case proposed osimertinib as a reliable treatment option. To date, all studies of BC with the EGFR T790M mutation, including this one, are case reports based on the genomic status of individual patients. The clinical value of the population of breast cancer patients with EGFR T790M mutations should be systematically evaluated in larger cohorts. Nevertheless, additional preclinical studies and clinical evidence are needed to increase our understanding of this area, and multidisciplinary discussions on individualized management are also required (1).

## 4 Conclusion

In summary, we report a patient with MMPMNs harboring a primary EGFR T790M mutation in BC tissue. This rare case proposes a reliable treatment option for BC with EGFR T790M mutation and highlights the importance of clinical actionability derived from comprehensive genomic profiling results outside standard treatment strategies. The diagnosis and treatment of BC patients with rare genetic mutations remain challenging because of the lack of specific screening and well−established treatment guidelines.

## Data availability statement

The original contributions presented in the study are included in the article/**Supplementary Material**. Further inquiries can be directed to the corresponding author.

## Ethics statement

Written informed consent was obtained from the individual(s) for the publication of any potentially identifiable images or data included in this article.

## Author contributions

All authors made substantial contributions to conception and design, acquisition of data, or analysis and interpretation of data; took part in drafting the article or revising it critically for important intellectual content; gave final approval of the version to be published; and agree to be accountable for all aspects of the work.
