# Supplementary material for: Next-generation sequencing-based detection in a breast MMPMN patient with EGFR T790M mutation: a rare case report and literature review
Source: Front Oncol. 2023 Jul 24;13:1204041. doi: 10.3389/fonc.2023.1204041 (PMC10405930; doi:10.3389/fonc.2023.1204041)
Supplement: Supplementary file 1 [file DataSheet_1.pdf]

Supplementary Table S1. Gene list of **425** cancer-related genes.

*ABCB1 (MDR1), ABCB4, ABCC2 (MRP2), ADH1A, ADH1B, ADH1C, AIP, AKT1, AKT2, AKT3, ALDH2, ALK, AMER1, APC, AR, ARAF, ARID1A, ARID1B, ARID2, ARID5B, ASCL4, ASXL1, ATF1, ATIC, ATM, ATR, ATRX, AURKA, AURKB, AXIN2, AXL, B2M, BAD, BAI3, BAK1, BAP1, BARD1, BAX, BCL2, BCL2L11 (BIM), BCR, BIRC3, BLM, BMPR1A, BRAF, BRCA1, BRCA2, BRD4, BRIP1, BTG2, BTK, BUB1B, CLLORF30, CASP8, CBL, CBLB, CCND1, CCNE1, CD274 (PD-L1), CD74, CDA, CDC73, CDH1, CDK10, CDK12, CDK4, CDK6, CDK8, CDKN1A, CDKN1B, CDKN1C, CDKN2A, CDKN2B, CDKN2C, CEBPA, CEP57, CHD4, CHEK1, CHEK2, CREBBP, CRKL, CSF1R, CTCF, CTLA4, CTNNB1, CUL3, CUX1, CXCR4, CYLD, CYP19A1, CYP2A13, CYP2A6, CYP2A7, CYP2B6*6, CYP2C19*2, CYP2C9*3, CYP2D6, CYP3A4*4, CYP3A5, DAXX, DDR2, DENND1A, DHFR, DICER1, DLL3, DNMT3A, DPYD, DUSP2, EGFR, EML4, EP300, EPAS1, EPCAM, EPHA2, EPHA3, EPHA5, EPHB2, ERBB2 (HER2), ERBB2IP, ERBB3, ERBB4, ERCC1, ERCC2, ERCC3, ERCC4, ERCC5, ESR1, ETV1, ETV4, ETV6, EWSR1, EXT1, EXT2, EZH2, FANCA, FANCC, FANCD2, FANCE, FANCF, FANCG, FANCI, FANCL, FANCM, FAT1, FBXW7, FGF19, FGFR1, FGFR2, FGFR3, FGFR4, FH, FLCN, FLT1 (VEGFR1), FLT3, FLT4, FOXA1, FOXP1, FRG1, GATA1, GATA2, GATA3, GATA4, GATA6, GNA11, GNAQ, GNAS, GRIN2A, GRM3, GRM8, GSTM1, GSTM4, GSTM5, GSTP1, GSTT1, HDAC2, HDAC9, HGF, HLA-A, HNF1A, HNF1B, HRAS, HSD3B1, IDH1, IDH2, IFNG, IFNGR1, IGF1R, IGF2, IKBKE, IKZF1, IL7R, INPP4B, IRF2, JAK1, JAK2, JAK3, JARID2, JUN, KDM5A, KDM6A, KDR (VEGFR2), KEAP1, KIF1B, KIF5B, KIT, KITLG, KLLN, KMT2A (MLL), KMT2B, KMT2C, KMT2D, KRAS, LHCGR, LMO1, LRP1B, LYN, LZTR1, MAP2K1 (MEK1), MAP2K2 (MEK2), MAP2K4, MAP3K1, MAP3K4, MAP4K3, MAX, MCL1, MDM2, MDM4, MECOM, MED12, MEF2B, MEN1, MET, MGMT, MITF, MLH1, MLH3, MLLT1, MLLT3, MLLT4, MPL, MRE11A, MSH2, MSH6, MTHFR, MTOR, MUTYH, MYC, MYC, MYCN, MYD88, MYH9, NAT1, NBN NCOR1, NF1, NF2, NFE2L2, NFKBIA, NKX2-1, NKX2-4, NOTCH1, NOTCH2, NOTCH3, NPM1, NQO1, NRAS, NRG1,*

NSD1, NTRK1, NTRK2, NTRK3, PAK3, PALB2, PALLD, PARK2, PARP1, PARP2, PAX5, PBRM1, PDCD1 (PD1), PDCD1LG2 (PD-L2), PDE11A, PDGFRA, PDGFRB, PDK1, PGR, PHOX2B, PIK3C3, PIK3CA, PIK3R1, PIK3R2, PKHD1, PLAG1, PLK1, PMS1, PMS2, POLD1, POLD3, POLE, POLH, POT1, PPARD, PPP2R1A, PRDM1, PRF1, PRKACA, PRKACG, PRKAR1A, PRKCI, PRKDC, PRSS1, PRSS3, PTCH1, PTEN, PTK2, PTPN11, PTPN13, PTPRD, QKI, RAC1, RAC3, RAD50, RAD51, RAD51B, RAD51C, RAD51D, RAD54L, RAF1, RARA, RARG, RASGEF1A, RB1, RECQL4, RELN, RET, RHOA, RICTOR, RNF43, ROS1, RPTOR, RRM1, RUNX1, RUNX1T1, SBDS, SDC4, SDHA, SDHB, SDHC, SDHD, SEPT9, SETBP1, SETD2, SF3B1, SGK1, SLC34A2, SLC3A2, SLC7A8, SMAD2, SMAD3, SMAD4, SMAD7, SMARCA4, SMARCB1, SMO, SOS1, SOX1, SOX14, SOX2, SOX21, SPOP, SPRY4, SRC, SRY, STAG2, STAT3, STK11, STMN1, STT3A, SUFU, TAP1, TAP2, TEK, TEKT4, TERC, TERT, TET2, TGFBR2, THADA, TMEM127, TMPRSS2, TNFAIP3, TNFRSF11A, TNFRSF14, TNFRSF19, TNFSF11, TOP1, OP2A, TP53, TP63, TPMT, TSC1, TSC2, TSHR, TTF1, TUBB3, TUBB4A, TUBB4B, TUBB6, TYMS, U2AF1, UGT1A1, VAMP2, VEGFA, VHL, WAS, WISP3, WRN, WT1, XPA, XPC, XRCC1, YAP1, ZNF2, ZNF217, ZNF703

Supplementary Table S2. The allele frequencies of genetic alterations detected by targeted NGS Sequencing in different sample types.

Gene	Alteration	Mutation type	Sample type			
			Plasma ctDNA	Ovary tissue (FFPE)	Breast tissue (FFPE)	Axillary lymph node tissue (FFPE)
EGFR	p.T790M	c.2369C>T	-	-	1.59%	-
TP53	p.Y220C	c.659A>G	3.36%	-	-	75.65%
NBN	p.R670*	c.2008_2009delinsT	0.11%	2.73%	2.10%	-
MPL	p.S566Rfs*82	c.1698_1840del	-	7.21%	8.25%	-
NOTCH1	p.Y527*	c.1581C>G	-	4.23%	3.62%	-
ARID1A	p.P94S	c.280C>T	0.67%	-	-	29.15%
ARID1B	p.S640C	c.1919C>T	-	0.57%	-	-
BRCA1-2			-	-	-	-

FFPE: formalin-fixed, paraffin-embedded; “-”: not detected; ctDNA: circulating tumor DNA.
