# Supplementary figures and images for: Next-generation sequencing-based detection in a breast MMPMN patient with EGFR T790M mutation: a rare case report and literature review
Source: Front Oncol. 2023 Jul 24;13:1204041. doi: 10.3389/fonc.2023.1204041 (PMC10405930; doi:10.3389/fonc.2023.1204041)

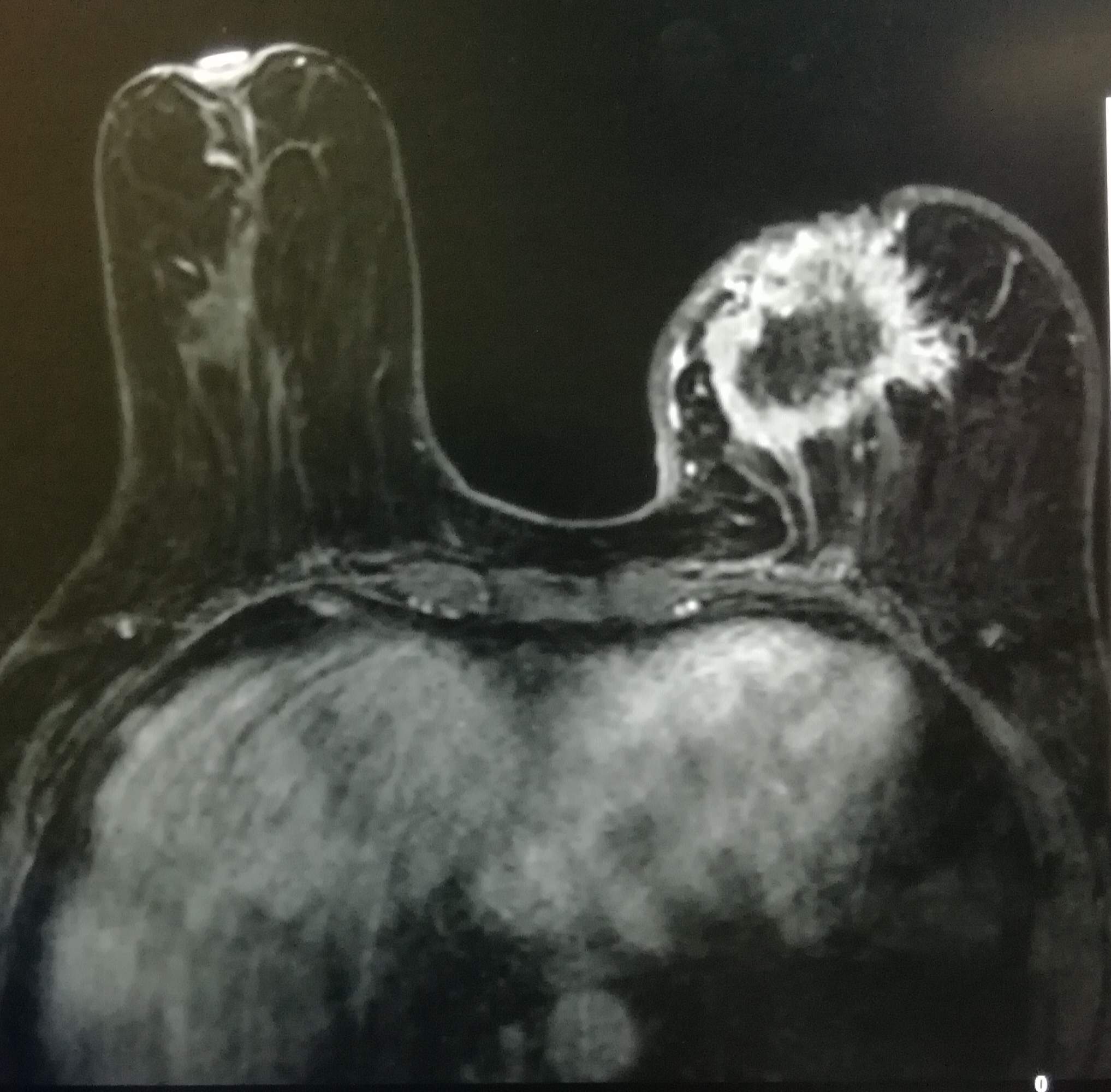

Supplement: Supplementary file 2 [file Image_1.jpeg]
